# Video observation of hand hygiene practices during routine companion animal appointments and the effect of a poster intervention on hand hygiene compliance

**DOI:** 10.1186/1746-6148-10-106

**Published:** 2014-05-07

**Authors:** Maureen EC Anderson, Jan M Sargeant, J Scott Weese

**Affiliations:** 1 Department of Pathobiology, University of Guelph, Guelph, ON, Canada; 2 Department of Population Medicine, University of Guelph, Guelph, ON, Canada

**Keywords:** Veterinary, Companion animal, Hand hygiene, Infection control, Video observation, Intervention

## Abstract

**Background:**

Hand hygiene is considered one of the most important infection control measures in human healthcare settings, but there is little information available regarding hand hygiene frequency and technique used in veterinary clinics. The objectives of this study were to describe hand hygiene practices associated with routine appointments in companion animal clinics in Ontario, and the effectiveness of a poster campaign to improve hand hygiene compliance.

**Results:**

Observation of hand hygiene practices was performed in 51 clinics for approximately 3 weeks each using 2 small wireless surveillance cameras: one in an exam room, and one in the most likely location for hand hygiene to be performed outside the exam room following an appointment. Data from 38 clinics were included in the final analysis, including 449 individuals, 1139 appointments before and after the poster intervention, and 10894 hand hygiene opportunities. Overall hand hygiene compliance was 14% (1473/10894), while before and after patient contact compliance was 3% (123/4377) and 26% (1145/4377), respectively. Soap and water was used for 87% (1182/1353) of observed hand hygiene attempts with a mean contact time of 4 s (median 2 s, range 1-49 s), while alcohol-based hand rub (ABHR) was used for 7% (98/1353) of attempts with a mean contact time of 8 s (median 7 s, range 1-30 s). The presence of the posters had no significant effect on compliance, although some staff reported that they felt the posters did increase their personal awareness of the need to perform hand hygiene, and the posters had some effect on product contact times.

**Conclusions:**

Overall hand hygiene compliance in veterinary clinics in this study was low, and contact time with hand hygiene products was frequently below current recommendations. Use of ABHR was low despite its advantages over hand washing and availability in the majority of clinics. The poster campaign had a limited effect on its own, but could still be used as a component of a multimodal hand hygiene campaign. Improving the infection control culture in veterinary medicine would facilitate future campaigns and studies in this area, as well as overall patient and staff safety.

## Background

Hand hygiene is a critical infection control measure for the prevention of hospital-associated infections (HAIs) in human healthcare settings [1,2]. In various jurisdictions, hospitals are now required to collect and report hand hygiene compliance data as part of programs to help improve patient safety and quality of care, as well as ensure facility accountability [3-5]. In veterinary medicine, infection control guidelines also recommend proper hand hygiene practices as one of the most important measures for reducing the spread of pathogens in clinics [6-8]. However, hand hygiene compliance in veterinary clinics has received little attention, and only recently have a small number of studies begun to investigate the actual use of this simple yet essential infection control practice amongst veterinary staff, reporting compliance of 21-48% in companion animal personnel [9-11].

The gold standard for measuring hand hygiene compliance in human healthcare facilities remains direct observation by individuals on the clinic floor [2,12-14]. In most primary care veterinary clinics, where there is typically a relatively small number of staff and a substantial amount of animal contact takes place during outpatient appointments in private exam rooms, the presence of a designated hand hygiene observer would likely be considered unacceptably cumbersome and intrusive, and result in significant bias due to Hawthorne effects (whereby individuals may alter their behaviour because they know they are being observed [15]). The use of video cameras for direct observation does not eliminate the potential for Hawthorne effects, but can reduce it substantially, as observations can be made considerably more discretely. Even in fixed positions, cameras can capture a substantial amount of raw data when strategically used in a relatively limited environment, such as an exam room or treatment area. Video observation of hand hygiene practices is not frequently used in healthcare settings [16], but has been used successfully in food handling studies [17,18].

Based on the low staff hand hygiene compliance reported in recent veterinary studies, the effectiveness of measures to improve compliance warrants investigation. Posters are a commonly used type of intervention for promoting a wide variety of ideas and behaviours in many settings, and they are often incorporated into multimodal interventions for improving hand hygiene compliance in healthcare facilities [19-21]. The impact of hand hygiene poster campaigns is variable and may be short-term, and often cannot be separated from the effect of concurrent interventions [22,23]. Careful attention to the design of the campaign message and the poster(s) is critical, but thereafter implementation of a basic poster campaign is fairly simple and requires little to no effort on the part of the targeted individuals. This makes a hand hygiene poster intervention a reasonable “first step” in facilities that may be resistant to more involved, active interventions due to lack of a strong infection control culture or other reasons.

The objectives of this study were to use video observation to describe hand hygiene compliance in terms of timing and technique associated with routine appointments in primary care companion animal clinics in Ontario, as well as to evaluate the effectiveness of a basic poster campaign to improve hand hygiene compliance, and to identify factors that could potentially help guide the design and implementation of future interventions to improve hand hygiene practices in veterinary clinics.

## Methods

### Clinic recruitment

A convenience sample of primary care companion and mixed animal veterinary clinics from across southwestern and eastern Ontario, Canada, was recruited to participate. Clinics in various regions were identified through known contacts of two of the authors (MA or JW) and using Google Maps [maps.google.ca] with the search term “veterinary”. Each clinic was then contacted directly by one of the investigators via e-mail, fax or telephone, typically obtained from individual clinic websites. If no response was received, follow-up inquiries were made by the same means 1, 3 and 5 weeks later, and then monthly thereafter until recruitment was complete. Data collection was performed from November 2010 to December 2011.

### Video observation

Two wireless video surveillance cameras (Logitech WiLife™ Indoor Video Security System, Logitech, Newark, CA) were installed in each clinic: one in an exam room, and one in the most likely backroom location for hand hygiene to be performed outside the exam room following an appointment (excluding private offices and washrooms), as determined by clinic layout and information on clinic workflow provided by staff. The cameras were visible to staff, but care was taken to position the cameras and secure their power cords to make them as discrete as possible. All indicator lights on the cameras were disabled so there were no visible signs that the cameras were on or off. Video data were recorded by powerline network on a secure, closed laptop computer kept elsewhere in the clinic in an unobtrusive location (e.g. on top of a cupboard, under a desk, on an unused shelf), using the software provided by the camera manufacturer (Logitech Command Center v2.5 (for Windows), Logitech, Newark, CA). Cameras were left in place for 14-19 working days (19-23 calendar days), and were motion-activated during the hours when routine (non-emergency) appointments were typically scheduled in each clinic, plus approximately 30 min before and after this period. The cameras did not record audio data.

### Poster intervention

Two different poster designs (A and B) were used in each clinic [see Additional files 1 and 2]. Key elements of the posters included gain-framing of messages, emphasis on minimization of losses/barriers, reminders of personal applicability, and appeal to obligation to protect others. The justification for these and other elements of both posters is detailed in an additional table [see Additional file 3]. A copy of Poster A (22 cm × 28 cm) was displayed in every exam room (monitored and unmonitored) in a location intended to be highly visible to staff during the appointment, such as over the sink (if present), close to the computer work station (if present) or at the door. Poster B (28 cm × 22 cm) was displayed near up to 3 hand hygiene stations (most often sinks) in the backroom area, particularly those closest to the monitored exam room and most likely to be used by staff for hand hygiene between appointments. All posters were mounted by one investigator (MA) between 9-13 working days after the cameras were installed. The posters did not include any information pertaining to non-hand hygiene infection control measures.

### Participant consent

Written consent was obtained from all clinic personnel whose images would potentially be captured on video; they were informed that the focus of the study was general infection control practices, but not for what specific practices data would be collected (including hand hygiene). Consent was not obtained from clients, as per the approved study protocol. This study was approved by the University of Guelph Research Ethics Board.

### Follow-up survey

At the end of the study, during the final site visit, an anonymous, voluntary written survey was provided to up to 20 staff members at each clinic. Surveys were provided in a pre-addressed, postage-paid envelope, and the primary staff contact at each clinic (veterinarian, technician or office manager) was asked to distribute them to the other staff and return any completed or uncompleted questionnaires within two weeks, at which time an email or telephone reminder was sent if the envelope had not yet been received. Any staff member who worked in the clinic during the video observation period was eligible to participate, but a maximum of 20 individuals from any one clinic were allowed to fill out the survey. Distribution and collection of the surveys was ultimately at the discretion of the clinic staff, including at clinics with over 20 staff members. The first five questions on the survey queried individual perception of and response to the poster intervention, in the form of yes/no and Likert-type scale questions. The remainder of the survey consisted of questions regarding hand hygiene practices and perceptions in general [24].

### Video coding - scheme

A video coding scheme was developed in the form of a fillable spreadsheet (Excel 2008 for Mac, Microsoft Corporation, Redmond, WA) and tested using video recordings from two clinics that were excluded from the final analysis due to loss of data from computer malfunctions. The scheme was modified from the World Health Organization’s (WHO) “5 moments for hand hygiene” [13,25]. The five types of hand hygiene opportunity used in this study were: (1) before animal contact, (2) before a “clean” procedure (with or without gloves), (3) after a “dirty” procedure without gloves, (4) after glove removal, (5) after animal contact. Table 1 lists common procedures that were considered “clean” or “dirty”. Timing of hand hygiene attempts was coded as: (0) unobserved (i.e. individual left field of view), (1) not performed (i.e. no attempt prior to (next) “clean” procedure, contact with a “cleaner” part of the same animal, or contact with an unrelated animal), (2) outside of room after touching other objects/surfaces, (3) outside of room before touching other objects/surfaces, (4) in room after touching other objects/surfaces not in direct contact with the animal, (5) in room before touching other objects/surfaces not in direct contact with the animal. The information that was coded at the clinic, appointment, individual and hand hygiene opportunity levels, respectively, is described in an additional file [see Additional file 4].

**Table 1:** Procedures in all species considered “clean” or “dirty” for hand hygiene monitoring in veterinary clinics

**Clean procedures:**	**Dirty procedures:**
Those more likely to result in contamination of sterile or privileged body sites/tissues with potentially infectious microbes carried on the hands	Those more likely to result in contamination of the hands with potentially infectious microbes from the patient or clinical specimens
-Injections (including but not limited to subcutaneous, intramuscular, intravenous)^1^	-Ear swabs and/or ear cleaning^2^
-Venipuncture (any vein)	-Digital rectal exam and/or expression of anal glands^3^
-Fine needle aspirate (including but not limited to cystocentesis, arthrocentesis, abdominocentesis, aspiration of masses, aspiration of lymph nodes)	-Removal of an old/dirty bandage from over a skin lesion/wound/incision
-Direct contact with a surgical incision (including suture removal)	-Cleaning and/or debridement of a skin lesion/wound/incision
-Application of a new/clean bandage over a skin lesion/wound/incision	-Abscess drainage or other contact with pus
-Application of solution or ointment to the eye (including fluorescein stain)	-Any contact with feces
-Placement (but not removal) of acupuncture needles	-Manipulation inside an animal’s mouth^4^
	-Skin scrapings

^1^injection of any substance into an animal to facilitate or carry out euthanasia was not considered a clean procedure.

^2^direct application of ear medication/drops was not considered a dirty procedure if no material/fluid was subsequently removed from the ear canal.

^3^use of a rectal thermometer was not considered a dirty procedure.

^4^manipulation inside an animal’s mouth included direct administration of pills (i.e. not using a pilling wand, syringe or any means by which the animal consumed the medication voluntarily (e.g. hidden in food)); examination of the buccal gingiva if the fingers were not placed in the buccal sulcus or further into the mouth was not considered a dirty procedure.

The availability of alcohol-based hand rub (ABHR) in each clinic was determined by observation of ABHR dispensers in high-traffic areas during site visits (camera set up, poster mounting, camera take-down), particularly in the monitored areas (exam room, backroom). Hand hygiene product contact time was measured in seconds from the moment the product came into contact with both hands (i.e. start of scrubbing/rubbing) to the moment when contact with running water ceased (if water only used) or when contact with running water for rinsing began (if soap and water used) or when rubbing ceased (if ABHR used). If an individual applied ABHR but left the field of view before ceasing hand rubbing, the attempt was coded without a product contact time and without a hand drying technique. A technique score from 0 to 4 was produced for each hand hygiene attempt for which contact time was > 1 s and all four technique variables (i.e. deliberate effort to scrub/rub back of hands, between fingers, thumbs, and wrists) were coded as either yes or no, with one point given for each component when scrubbing/rubbing was observed. Additional details of the video coding scheme are described in an additional file [see Additional file 5].

### Video coding - process

All videos were coded by the same author (MA). Consecutive appointments were coded from the time the hand hygiene posters were placed in the clinic for a maximum of 8 days (i.e. end of recording) or up to 40 appointments, and then an equal number of appointments was coded working backward from the same point, for a maximum of 80 appointments per clinic, not including incomplete appointments. This number was selected in order to maximize the amount of data that could be included from each clinic while avoiding excessive representation of very busy clinics in the data set, based on an estimate that on average 8-10 appointments would be seen per day in a single exam room over two weeks. An appointment was defined as outpatient care provided to one or more animals presented to the clinic by an individual guardian during a single visit on a given day, beginning and/or ending in the monitored exam room. An appointment was classified as incomplete if a segment of the video footage from the exam room was missing (e.g. if the appointment started before or ended after the cameras were scheduled to be on, or if there was a problem with the relay to the computer) which potentially included a hand hygiene attempt prior to an individual’s first contact with the patient or following an individual’s final contact with the patient. For each incomplete appointment an additional complete consecutive appointment was coded, and incomplete appointments were excluded from the analysis. It was considered unlikely that there was any association between the classification of an appointment as incomplete and the likelihood of hand hygiene compliance or product contact times during the appointment; however, the potential for some selection bias due to this process cannot be entirely ruled out. The minimum data set required for inclusion of a clinic was 10 appointments pre- and post-intervention, respectively.

Videos were generally scanned at 2-4 times normal speed, and then watched in real time or slow motion with repeated review as necessary to discern pertinent actions. All procedures of interest, including related hand hygiene opportunities, captured on either the exam room or backroom video, and associated with an appointment initiated in the monitored exam room, were coded. Procedures captured on video that were related to inpatients or outpatients seen in other exam rooms were not coded.

### Statistical analysis

Coded data were imported into a statistical software package (SAS 9.3, SAS Institute Inc., Cary, NC) for analysis. Descriptive statistics were examined for all dependent and independent variables. Data for contact time with hand hygiene product were not normally distributed; therefore, a log transformation of contact time was used for modeling. Two separate multivariable statistical models were constructed: one for observation of a hand hygiene attempt in either monitored area (exam room or backroom) using mixed logistic regression via the Proc GLIMMIX procedure (hand hygiene compliance model), and one for the log of hand hygiene product contact time using mixed linear regression via the Proc Mixed procedure (contact time model). Variables of interest tested in both models, which were determined *a priori*, were: role, gender, sink in exam room, ABHR readily available in clinic, presence of posters, recording day (as a continuous variable), appointment type, room, species, and hand hygiene opportunity type. Hand hygiene product used was also tested in the contact time model. Random effect terms for clinic, appointment (grouped by clinic) and individual (grouped by clinic and appointment) were initially added to both models to account for the potential impact of clustering by each of these variables.

A backward stepwise selection approach was used for model construction. Significance was set at p ≤ 0.05. All variables of interest were initially included in the model, then variables with a p > 0.05 were removed, one at a time starting with the largest p-value, noting the effect of removal on the coefficients for the remaining variables. If at least 1 of the coefficients for the remaining variables changed by more than 20%, the eliminated variable was deemed to be a confounder and restored to the model. Presence of posters was forced into the final models, as this was a primary variable of interest. This process generated the “main effects model.” Biologically plausible 2-way interactions between variables in the main effects model were assessed and if the p-value was ≤0.05 the term was retained in the model. Variables that were part of a significant interaction term were retained in the final model regardless of their individual significance.

For the contact time model, residuals for observations at the hand hygiene opportunity level were examined graphically using scatter plots, histograms and normal quantile plots to assess normality, and to identify potential outlier observations and variables with unequal variance. Outliers were further examined to ensure they were not the result of errors in data entry. Normality of residuals was also assessed using the Anderson-Darling and Shapiro-Wilk tests.

## Results

A total of 135 clinics were approached to participate in the study, out of approximately 1100 registered companion animal hospitals in Ontario (12%). Of these, no response of any kind was received from 26 (19%). Fifty-seven clinics (42%) declined to participate for the following reasons: staff not comfortable with the cameras (13), concern regarding clients being uncomfortable with the cameras (4), too busy and/or undergoing renovations (5), new personnel on staff (3), and no reason given (32). Fifty-two clinics (38%) agreed to participate, one of which was excluded as it was determined that the caseload was primarily emergency. Of the 51 clinics in which the video monitoring was performed, one was excluded due to staffing issues and plumbing problems in one of the monitored areas, and one because signs were posted by clinic staff next to the cameras alerting personnel and clients to their presence. Eleven other clinics were excluded due to lack of sufficient data to complete a minimum data set due to the following reasons: power failures (4), technical issue with the programming of the camera schedule (3), inadequate number of appointments (2), computer memory error (1), limited recording based on the work schedule of a single technician who did not wish to participate in the study (1). Ultimately videos from 38 facilities were coded and included in the analysis for this study, all of which were exclusively companion animal clinics.

Data from 2278 appointments were coded, including 887 (39%) appointments during which vaccine administration was observed. Sixty-seven percent (1532/2278) of appointments involved a single dog, 24% (542/2278) involved a single cat, 8% (190/2278) involved multiple dogs and/or cats, and 0.6% (14/2278) involved an animal of another species (e.g. rabbit, bird, rodent). Sixty-six (3%) appointments were classified as incomplete, with a maximum of 7 or up to 9% of appointments in any single clinic. The number of complete appointments coded per clinic ranged from 20-80 (mean 60, median 67). A total of 10894 hand hygiene opportunities were observed, involving approximately 449 unique individuals. The number of individuals coded per clinic ranged from 4-39 (mean 12, median 11), and the number of hand hygiene opportunities per clinic ranged from 70-631 (mean 287, median 285). Selected descriptive data for each of the 38 clinics can be found in Additional file 6. Thirteen percent (58/449) of participants were male, of which 67% (39/58) were veterinarians, 17% (10/58) were technicians and 16% (9/58) were other support staff (e.g. receptionists, students, volunteers). Of 391 female participants, 22% (86/391) were veterinarians, 59% (229/391) were technicians, and 19% (76/391) were other support staff.

**Figure 1:**
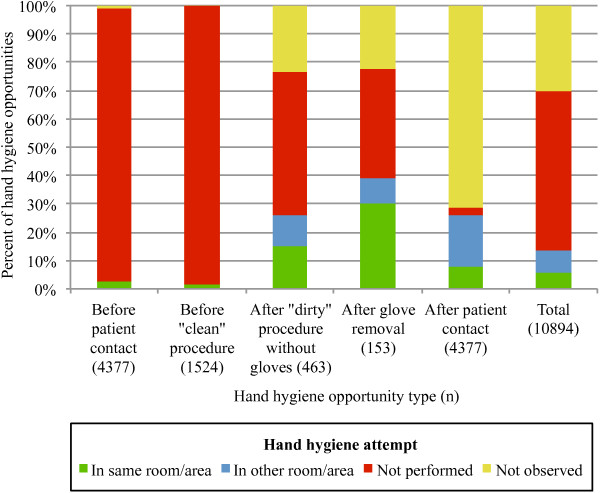
**Hand hygiene opportunities and attempts observed during 2278 companion animal veterinary appointments.** See Table 1 for list of procedures considered “clean” vs “dirty”. These data are presented in more detail in tabular format in Additional file 7.

Figure 1 shows the distribution of hand hygiene opportunities by type and whether a hand hygiene attempt was observed, not performed or not observed. Overall hand hygiene compliance (calculated as total number of opportunities for which a hand hygiene attempt was observed divided by total number of opportunities observed) was 14% (1473/10894), and ranged from 1-28% (mean 13%, median 12%) in individual clinics. Compliance was highest after glove removal (39%, 60/153) followed by after patient contact (26%, 1145/4377), after a “dirty” procedure without gloves (26%, 120/463), before patient contact (3%, 123/4377) and was lowest before a “clean” procedure (2%, 25/1524). Of the 3201 opportunities after which the individual left the room/area where the contact/procedure took place and a hand hygiene attempt was not observed, in 48% (1545/3201) the individual left the field of view without touching any objects/surfaces. For 1% (97/10894) of opportunities, an off-camera hand hygiene station was available in the immediate area (in the exam room in one clinic (86 opportunities) and in the backroom in another clinic (11 opportunities)) and it could not be determined from the footage if hand hygiene was attempted in the room/area or not.

Timing of glove removal was observed in 152 instances. Of these, in 62% (94/152) gloves were removed in the room before having contact with a “cleaner” part of the animal or other objects/surfaces not in direct contact with the animal, in 29% (44/152) gloves were removed in the room but after having contact with other objects/surfaces not in direct contact with the animal, in 4% (6/152) gloves were removed after leaving the room before contact with objects/surfaces in the next room, and in 5% (8/152) the individual left the room and returned still wearing the same pair of used/dirty gloves. Anecdotally, gloves were most commonly worn to perform digital rectal exams and/or to express anal glands of patients, and it was common for individuals to don only one glove for such a procedure.

A sink was present in the exam room in 66% (25/38) of clinics, and ABHR was readily available in 68% (26/38) of clinics. Eleven percent (4/38) of clinics had neither a sink in the exam room nor ABHR readily available [see Additional file 6]. A total of 1353 hand hygiene attempts were observed, accounting for 1246 unique hand hygiene opportunities, 227 coincident hand hygiene opportunities (in which a single attempt fulfilled more than one hand hygiene indication/opportunity), and 14% (1473/10894) of all hand hygiene opportunities. Soap and water was used for 87% (1182/1353) of all attempts, water alone for 5% (63/1353), and for 1% (10/1353) water was used but it was not possible to determine if soap was used as well. Use of bar soap was not observed, only liquid soap. Alcohol-based hand rub was used in 7% (98/1353) of all hand hygiene attempts, and in 1% (98/8078) of opportunities and 10% (98/990) of attempts that occurred in clinics where ABHR was readily available. In no instance in which ABHR was used for hand hygiene were the hands of the individual grossly contaminated to the point that ABHR would be contraindicated, based on lack of visible gross contamination on the video, the level of gross contamination that would be anticipated based on the procedure performed, or both. Descriptive statistics for hand hygiene product contact times are shown in Table 2.

**Table 2:** Contact times with hand hygiene products observed during 1343 hand hygiene attempts in veterinary clinics

		**Product contact time (s)**					
**HH product**	**Percent of HH attempts observed (n)**	**n**^**1**^	**Mean**	**Q1**	**Q2**	**Q3**	**Range**
Water alone	5 (63)	63	2	1	2	2	1-11
Soap	88 (1182)	1180	4	1	2	6	1-49
ABHR	7 (98)	87	8	3	7	13	1-30

HH = hand hygiene, ABHR = alcohol-based hand rub, Q = quartile.

^1^denotes the number of HH attempts for which contact time was measurable based on the available video.

Observation of hand hygiene technique in terms of deliberate effort to scrub/rub various parts of the hands (i.e. backs, between fingers, thumbs, wrists) was often difficult based on camera angle, visual obstructions and in some cases video resolution. For 38% (509/1353) of hand hygiene attempts, contact time with the product used was 1 s, which was considered inadequate to deliberately scrub/rub any part of the hands. Of the remaining 844 attempts observed, coding for all four technique variables was complete (i.e. no variable was coded as “not visible”) in 45% (379/844), representing 92% (35/38) of clinics. Scrubbing/rubbing between fingers was most commonly observed in 30% (113/379) of attempts, followed by back of hands in 27% (101/379), thumbs in 13% (49/379) and wrists in 4% (15/379). The technique scores produced for these hand hygiene attempts using water alone, soap and ABHR are shown in Table 3.

**Table 3:** **Hand hygiene technique scores**^**1 **^**for 379 hand hygiene attempts with product contact times >1 s**

		**HH technique score**				
**HH product**	**n**	**0**	**1**	**2**	**3**	**4**
		**% (n)**	**% (n)**	**% (n)**	**% (n)**	**% (n)**
Water alone	27	93 (25)	7 (2)	-	-	-
Soap	300	53 (158)	33 (100)	11 (32)	3 (8)	1 (2)
ABHR	52	13 (7)	33 (17)	40 (21)	13 (7)	-
Total	379	50 (190)	31 (119)	14 (53)	4 (15)	1 (2)

HH = hand hygiene, ABHR = alcohol-based hand rub.

^1^score was based on observation of deliberate effort to scrub/rub each of four separate parts of the hands (backs, between fingers, thumbs, wrists), with one point given for each component, resulting in a score from 0-4.

Hand drying techniques observed are shown in Table 4. Single-use towels (e.g. paper towel) were the most common means of hand drying in 82% (31/38) of clinics. Use of reusable towels was seen in 34% (13/38) of clinics and was the most common means of hand drying in 18% (7/38). Direct hand contact with the water faucet after hand hygiene occurred in 99% (1223/1237) of attempts in which running water was used, including 19 instances of 3 different individuals in 2 separate clinics in which ABHR was used at a sink in place of soap. Use of paper towel to protect the hands when turning off the faucet was seen in 1% (9/1237) of attempts, and for all other attempts there was either no subsequent contact with the faucet (4) or part of the arm was used to turn the faucet off (1). Hand drying technique and faucet contact following use of running water could not be determined for 10 and 18 attempts, respectively. For 11% (11/98) of attempts using ABHR, the individual walked off camera while still rubbing, therefore neither total contact time nor drying technique were coded.

**Table 4:** Hand drying techniques observed for 1332 hand hygiene attempts in veterinary clinics

		**Hand drying technique**				
**Hand hygiene product used**	**n**	**None**	**Single-use/paper towel**	**Reusable/cloth towel**	**Wipe on clothes**	**Shake hands in air**
		**% (n)**	**% (n)**	**% (n)**	**% (n)**	**% (n)**
Water alone	63	-	90 (57)	10 (6)	-	-
Water +/- soap^1^	10	-	80 (8)	20 (2)	-	-
Soap	1172	0.1 (1)	76 (885)	24 (283)	0.1 (1)	0.2 (2)
ABHR	87	70 (61)	6 (5)	20 (17)	1 (1)	3 (3)
Total	1332	5 (62)	72 (955)	23 (308)	0.2 (2)	0.4 (5)

ABHR = alcohol-based hand rub.

^1^Hand hygiene was performed at sink with running water, but could not determine whether or not soap was used.

Approximately 74% (194/263) of personnel who were observed performing hand hygiene were seen wearing a ring, watch, bracelet (including any type of band or elastic worn around the wrist) or multiples of these items during patient contact. Of the 1353 hand hygiene attempts observed, a watch was worn for 13% (178/1353), one or more rings for 16% (221/1353), one or more bracelets for 4% (51/1353), a combination of more than one of these different items (most commonly a watch and ring) for 48% (643/1353) and no visible jewelry for 19% (260/1353). Removal of any jewelry prior to a hand hygiene attempt was noted on one occasion.

Direct face-to-animal contact or indirect facial contact via an individual’s hands occurred prior to hand hygiene in 60% (277/463) of opportunities after a “dirty” procedure, 49% (75/153) of opportunities after glove removal, and of 69% (3003/4377) of opportunities after regular contact with an animal during 96% (2191/2278) of all appointments.

### Quantitative analysis - hand hygiene compliance

The final model for hand hygiene compliance included terms for presence of posters (not significant, but forced in), recording day, room, role, gender, and opportunity type, and interaction terms for room*role, gender*opportunity type, and room*opportunity type (Table 5). Use of an interaction term for hand hygiene opportunity type and role resulted in the model failing to converge. The variables sink in the exam room, appointment type, ABHR readily available in clinic, and species were not significantly associated with compliance and were therefore not included in the final model. The final model was produced after 20 steps.

**Table 5:** P-values for variables in the final logistic regression model for HH compliance (n = 10894)

	**Term**	**P-value**
Fixed effects	Role	<0.0001
	Gender	0.0003
	Presence of posters	0.5347
	Recording day	0.0408
	Room	0.0103
	HH opportunity type	<0.0001
	Role*room	<0.0001
	Gender*HH opportunity type	0.0045
	Room*HH opportunity type	0.0007
Random effects	Clinic	0.0002
	Appointment (by clinic)	0.0152
	Individual (by clinic and appointment)	0.0764

HH = hand hygiene, *denotes an interaction term between the two variables listed.

**Figure 2:**
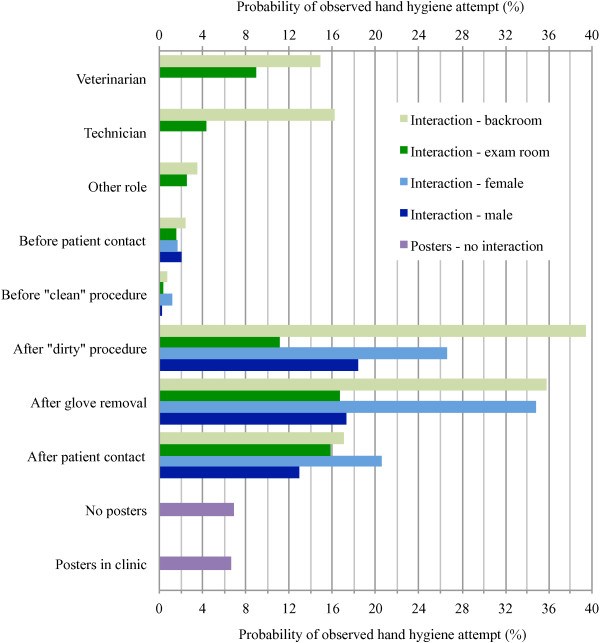
**Probabilities of hand hygiene compliance as per the final logistic regression model (n = 10894).** Interactions indicate the effect of the variables listed on the y-axis (e.g. role: veterinarian, technician, other role) differed according to the interaction variable (e.g. room: backroom vs exam room).

Adjusted probabilities of an observed hand hygiene attempt for each variable and combination of variables (for interactions) are shown in Figure 2. To see all significant differences refer to Additional file 8, which shows the odds ratios (ORs), 95% CIs and p-values for all possible contrasts of associations between variables in the final model. The effect of posters was not significant (p = 0.5347). The odds of an observed hand hygiene attempt increased by 1.04 for each recording day (95% CI 1.002-1.087, p = 0.0408). The effects of the other significant variables were all affected by interactions. In general, the odds of an observed hand hygiene attempt were higher for veterinarians and technicians compared to other staff, for opportunities after a procedure, glove removal or patient contact compared to before a procedure or patient contact, for females compared to males, and for opportunities observed in the backroom compared to the exam room.

### Quantitative analysis - hand hygiene product contact time

Based on descriptive statistics, data for hand hygiene product contact time were not normally distributed; therefore, a log transformation of contact time was used for modeling. Furthermore, the 10 observations for which it was unknown if soap and water or water alone was used were excluded from the contact time model.

The final contact time model included terms for role, gender, ABHR readily available in clinic, presence of posters, species, hand hygiene product, and opportunity type, and interaction terms for role*ABHR readily available in clinic, presence of posters*ABHR readily available in clinic, and presence of posters*opportunity type (Table 6). The variables recording day, sink in the exam room, appointment type and room were not significantly associated with contact time and were therefore not included in the final model. The estimated effect of clustering by appointment was consistently zero throughout the model-building process; therefore, this term was dropped from the final model. The final model was produced after 26 steps. Tests for normality (Anderson-Darling = 7.07, p < 0.005; Shapiro Wilk = 0.99, p < 0.0001) indicated that the residuals were not normally distributed; however, based on visual assessment of the histogram and normal quantile plot of the residuals, the distribution was considered adequate in terms of normality [see Additional file 9].

**Table 6:** P-values for variables in the final linear regression model for HH product contact time (n = 1330)

	**Term**	**P-value**
Fixed effects	Role	0.0017
	Gender	0.0120
	ABHR readily available in clinic	0.0004
	Presence of posters	0.0170
	Species	0.0348
	HH product	<0.0001
	HH opportunity type	0.0136
	Role*ABHR readily available in clinic	0.0002
	ABHR readily available in clinic*presence of posters	0.0211
	Presence of posters*HH opportunity type	0.0400
Random effects	Clinic	0.0003
	Individual (by clinic and appointment)	<0.0001

ABHR = alcohol-based hand rub, HH = hand hygiene, *denotes an interaction term between the two variables listed.

**Figure 3:**
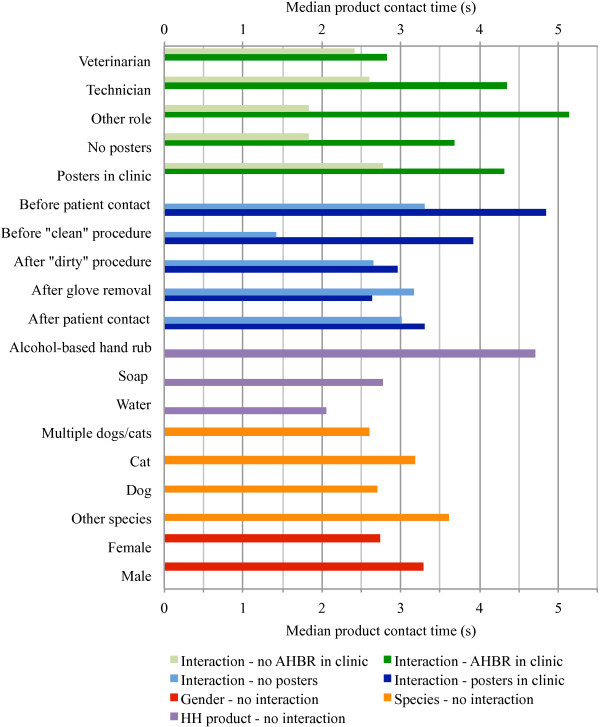
**Median hand hygiene product contact times as per the final linear regression model (n = 1330).** Interactions indicate the effect of the variables listed on the y-axis (e.g. role: veterinarian, technician, other role) differed according to the interaction variable (e.g. presence of AHBR: no AHBR in clinic vs AHBR in clinic).

Adjusted median values (geometric mean) for contact time for each variable and combination of variables (for interactions) are shown in Figure 3. To see all significant differences refer to Additional file 10, which shows the ratios of contact times, 95% CIs and p-values for all possible contrasts of associations between variables in the final model. Contact time for males was 1.20 times that for females (p = 0.0120, 95% CI 1.04-1.38). Contact time ratio was 1.18 for hand hygiene attempts associated with contact with individual cats compared to individual dogs (p = 0.0074, 95% CI 1.05-1.33), and 1.22 for individual cats compared to multiple animals (dogs and/or cats) in the same appointment (p = 0.0368, 95% CI 1.01-1.46). No other species comparisons reached statistical significance. Contact time ratio was 1.69 for ABHR compared to soap (p < 0.0001, 95% CI 1.38-2.08), 2.28 for ABHR compared to water alone (p < 0.0001, 95% CI 1.70-3.05), and 1.34 for soap compared to water alone (p < 0.0001, 95% CI 1.09-1.66). The effects of the other significant variables were all affected by interactions. In general, contact time was shorter for veterinarians compared to technicians and other staff in clinics where ABHR was readily available, and contact times were longer for hand hygiene attempts before patient contact or procedures when posters were present compared to attempts after procedures, glove removal or patient contact. Contact times were also longer in clinics where ABHR was readily available (independent of product used) but the difference was not significant for veterinarians. The presence of posters had a statistically significant effect on contact times in clinics where ABHR was not readily available (ratio of 1.50, p = 0.0043, 95% CI 1.14-1.99).

### Follow-up survey

**Figure 4:**
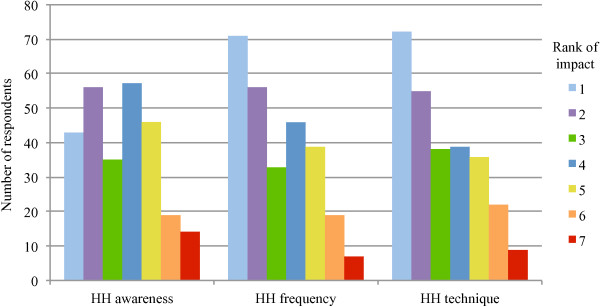
**Self-perceived impacts**^**1 **^**of a hand hygiene poster intervention on individual hand hygiene awareness and practices.**^1^Impact ranked on a scale of 1 (not at all) to 7 (very much). HH awareness = Posters increased awareness of need to perform hand hygiene and/or infection control in general (n = 270). HH frequency = Posters increased how often hand hygiene was performed (n = 271). HH technique = Posters increased how hand hygiene was performed (e.g. more thorough washing/rubbing) (n = 271).

Surveys were returned from 289 individuals (approximately 62% (289/465) of all staff to a maximum of 20 per clinic) from 37/38 clinics. The surveys from one clinic were mailed to the investigator but never received. Individual clinic response rate ranged from approximately 25-100% (mean 63%, median 60%). Survey respondents included 66 (23%) veterinarians, 71 (25%) registered veterinary technicians, 26 (9%) non-registered veterinary technicians, 25 (9%) animal care assistants/kennel staff, 80 (28%) front office staff, 15 (5%) practice managers, and 6 (2%) individuals who did not identify their primary role. Ninety-four percent (272/289) of respondents indicated that they noticed the hand hygiene posters that had been put up for the last week of the study. The self-perceived impacts of the posters on individual hand hygiene practices, ranked on a scale of 1 (not at all) to 7 (very much) are shown in Figure 4.

## Discussion

Observed hand hygiene compliance in this study (14%) was low compared to self-reported compliance among veterinary staff in other studies (42-48%) [10,11] and average reported compliance rates in human hospitals (40%) [12], but was comparable to (though still lower than) directly observed baseline hand hygiene compliance in a companion animal teaching hospital (21%), as recorded by live observers [9]. The lowest compliance by opportunity type was for before “clean” procedures, including all types of injections (e.g. vaccinations), which were a common occurrence. However, there are no existing recommendations to perform hand hygiene prior to vaccine administration in veterinary patients, even though hand hygiene has been recommended before administering injections in human medicine [25]. Hand hygiene prior to patient contact was 3%, compared to after patient contact and after “dirty” procedures at 26%, which was a significant difference in all contrasts in the multivariable model. This same finding, whereby healthcare workers tend to have better hand hygiene compliance after patient contact/procedures than before, has been reported in several other studies [15,26-28].

Based on the multivariable model, compliance was also better in the backroom compared to the exam room for both veterinarians and technicians, and for after “dirty” procedures. This could potentially be because staff feel they have more time or opportunity to perform hand hygiene when a client is not present; however, hand hygiene in the exam room has the added benefit of demonstrating commitment to infection control and providing a good example to pet owners, and therefore should be emphasized. The finding that veterinarians were significantly more likely to perform hand hygiene compared to technicians in the exam room is in contrast to studies in human medicine that have reported that nurses have better compliance than physicians [29,30]. Compliance among all staff who have contact with animals is important, but it appears to require the most improvement among other support staff, who consistently had the lowest compliance according to the model. The most statistically significant effect of gender was female staff were 1.75 times as likely to perform hand hygiene after patient contact than male staff. This is consistent with other studies that have identified male gender as a risk factor for decreased hand hygiene compliance and lower infection control “precaution awareness” [10,31].

The large number of opportunities for which a hand hygiene attempt was not observed also needs to be carefully considered. The likelihood that an individual could have performed hand hygiene in an unmonitored area varied considerably based on clinic layout and the availability of ABHR (as dispensers can be easily moved from place to place). Nonetheless, in each facility the monitored backroom area was considered the most likely station to be used during or between appointments based on location, convenience and staff routine; therefore, if hand hygiene was unobserved the individual would have had to bypass the two most likely stations to be used (assuming a hand hygiene station was present in the exam room), making it less likely that hand hygiene in relation to the appointment would be performed elsewhere.

The most commonly used product for hand hygiene in this study was liquid soap and water, including in clinics where ABHR was available. Although use of soap and water is recommended when hands are visibly soiled, or when an alcohol-resistant pathogen may be present (e.g. some non-enveloped viruses such as canine parvovirus, spore-forming bacteria such as *Clostridium* spp), use of ABHR is the primary recommendation for routine hand hygiene [2,8,12,32,33] as it is takes less time, causes less skin damage [12,34], can easily be used at the point-of-care even when a sink is not available, saves water, and generates less waste because disposable towels are not required for hand drying. The posters used in this study did not specify contraindications for the use of AHBR, but this would be an important component of any subsequent educational campaign or intervention. Hand hygiene using water alone was seen in a small percentage of attempts. Although simply rinsing hands with water can remove some superficial skin cells and loosely-adherent bacteria through mechanical flushing action [34], in the clinical setting the use of soap (either antimicrobial or non-antimicrobial depending on the specific situation) and water is recommended [8,12,33], and if hands are not adequately dried afterward rinsing alone may even increase the risk of pathogen transmission [35,36].

The presence of a sink in the exam room was not associated with observation of a hand hygiene attempt in the multivariable model when attempts in both the exam room and backroom were considered; nonetheless, it is very important to have sinks available in exam rooms and other patient care areas (e.g. treatment room, wards) in order to promote and facilitate hand hygiene, and to avoid the potential spread of microbes that may occur if an individual is forced to move to another area to find a hand hygiene station. Thirty-four percent of participating clinics in this study did not have a sink in the exam room. In these cases an ABHR dispenser can be placed in the room instead, and ideally procedures that are likely to result in gross contamination of the hands (and therefore require hand washing instead of ABHR) should be performed in another area where there is a sink. Effort should be made to facilitate use of the exam room sink as much as possible (e.g. by providing the desired soap product and ensuring sink is easily accessible and of adequate size), and staff should be encouraged to perform hand hygiene in the presence of clients.

In order to effectively reduce or kill the transient microbiota of the hands, a variety of sources recommended that soap be applied for a minimum of 10-20 s before rinsing, or for ABHR that enough product be applied to cover all surfaces of the hands and then rubbed until dry (which should also take at least 10-20s) [6,12,32,37-39]. The posters used in this study advocated 15 s contact time for both soap and ABHR, as this was consistent with a recent provincial public health campaign in Ontario [39]. However, the WHO Guidelines on Hand Hygiene, which are currently the definitive guidelines in human healthcare, recommend 40-60 s for complete hand washing (from wetting hands to completion of drying), and 20-30 for complete application of ABHR [2]. Product contact time was well below this range for the majority of hand hygiene attempts observed in this study (Table 2). Average duration of hand washing (i.e. the entire process, not just product contact time) by human healthcare workers in previous studies ranges from 5-24 s [12]. Contact times with ABHR were significantly longer than with soap; this may be in part due to the additional steps required to complete a hand wash (rinsing and drying) resulting in individuals abbreviating the scrubbing/contact time component of the process. With ABHR, more of this same time period can be devoted to the rubbing stage, which can also be done while moving to another area instead of standing at a sink. Improved contact times could be yet another reason to promote the use of ABHR in veterinary clinics. Misuse of ABHR was also seen. On several occasions various individuals were noted to use ABHR immediately after hand washing, which is not recommended [2,33]. Performance of rinsing or towel-drying after ABHR use should be discouraged, as should use of ABHR immediately after hand washing, as these unnecessary steps have the potential to curb contact times, and may result in additional damage to the skin, which can lead to increased carriage of pathogens on the hands and reluctance to perform hand hygiene subsequently due to discomfort [12,40].

Use of appropriate technique during hand hygiene is crucial to ensuring all parts of the hands come in contact with the product used and are adequately decontaminated. The areas most likely to be missed include the base of the thumbs, backs of the hands, between the fingers and beneath the fingernails [8,37,41]. Scrubbing/rubbing of wrists, which was observed the least often of the four selected technique components, could potentially be hindered by watches, bracelets or long sleeves, although this was not reported by another study [42]. Hand hygiene attempts with longer contact times would be more likely to achieve a higher technique score based on the four components, which may explain in part why scores tended to be higher for attempts using ABHR. It is unknown if or how much training veterinary personnel receive on hand hygiene technique. Some training, including emphasis on parts of the hands most often missed during hand hygiene, may be beneficial for improving technique. Improved technique would likely also result in increased product contact time.

Hand drying is an important component of hand hygiene, as bacteria are transferred from hands to surfaces much more readily when hands remain wet [35,36]. Single-use disposable paper towels are typically recommended for hand drying after hand washing in both the veterinary and human healthcare setting [2,6-8,12], and should be used to turn off manual water faucets to protect hands from immediate recontamination from the faucet handle(s) or knob(s). In this study, use of paper towel in this manner was seen after only 1% of hand hygiene attempts, and in some of these cases the individual continued to use the same paper towel to finish hand drying after turning off the faucet, thus negating the potential benefits of this practice. Reusable cloth towels are not recommended for use in the healthcare setting [2,12,36,37] due to their potential to act as a fomite between individual users. Nonetheless, reusable towels were used for hand drying in 23% of observed hand hygiene attempts in participating veterinary clinics, including after 20% of attempts using ABHR. Information on appropriate drying techniques and protecting hands from recontamination via the water faucet should be emphasized to veterinary personnel in training and educational campaigns.

Hand jewelry (e.g. watches, rings, bracelets) increases the number of microbes harbored on the hands [43]. The majority of veterinary personnel seen performing hand hygiene in this study wore at least one such item, and removal of hand jewelry prior to hand hygiene was rarely observed. While some studies have shown that jewelry such as rings can interfere with effective hand washing [44-46], no studies have shown an effect of rings on pathogen transmission via hands in the clinical setting [12]. The issue of whether or not the wearing of rings by healthcare workers should be allowed therefore remains unresolved [12]. There are very few studies examining the effect of wearing watches or bracelets on hand hygiene, with varying results [42,47]. While these items may or may not interfere with washing or sanitizing the hands and fingers, they have potential to interfere with these processes at the level of the wrist, and since they have been shown to increase bacterial load on the hands [43], some infection control guidelines recommend that these items be removed prior to patient contact or hand hygiene [7,8,32]. At a minimum, rings, watches and bracelets should be avoided if they are elaborate in design, or made from materials that absorb liquid and cannot be adequately cleaned if contaminated.

Gloves worn to perform “dirty” procedures should be removed immediately afterward, using an appropriate technique to avoid further contamination of the hands, and then hand hygiene should be performed [2,6,8,12]. In this study, gloves were removed in the same room/area in which they were used in 91% of cases, but in 29% of cases other objects or surfaces in the room were touched prior to glove removal, which has the potential to lead to environmental contamination and indirect transmission of pathogens to other people or animals. Failure to remove gloves prior to leaving the room/area (9% of cases) could lead to even wider-spread contamination, including common-touch surfaces such as doors. Hand hygiene compliance was actually highest after glove removal of all the hand hygiene opportunity types, and of the three “after” opportunity types hand hygiene was most frequently attempted in the same room (compared to the other room/area) after glove removal. Even so, observed hand hygiene compliance after glove removal was still only 39%. Glove use may be misconstrued as a substitute for hand hygiene, and has been reported as a barrier to hand hygiene in other studies [31,48,49]. Pre-existing defects or unnoticed damage to gloves during use, as well as the potential for contamination of the hands during glove removal make gloves an imperfect barrier [6,12,50], and the nature of the “dirty” procedures for which gloves are often worn makes hand hygiene following glove removal very important.

The coding scheme for hand hygiene opportunities used in this study was based largely on the WHO’s “5 moments for hand hygiene” [13,25]. An example of a typical appointment in which the patient is received first by a technician, and then examined by a veterinarian who also administers a vaccine would include 5 hand hygiene opportunities: before and after patient contact for each individual (moments 1 and 5), as well as before the “clean” procedure for the veterinarian (moment 2). There were many instances in which unnecessary or mistimed contact with an animal resulted in additional failed hand hygiene opportunities that could have been avoided, such as petting a patient at the end of an appointment after having just performed hand hygiene and then failing to do so again. Simple procedural changes could also potentially alleviate some of the need for additional hand hygiene attempts. For example, by performing “dirty” procedures at the end of an appointment, hand hygiene for after the procedure and after patient contact can be accomplished with the same attempt. Anecdotally, young puppies and kittens frequently had unnecessary contact with a number of staff, likely because they are considered “cute,” even though they may be at higher risk for pathogen transmission [38]. Although the additional social contact can be beneficial for the animal and is appealing to staff, if individuals are not prepared to perform appropriate hand hygiene then this sort of unnecessary contact should be avoided. Perfect hand hygiene compliance in any veterinary clinic based on this scheme may not be a realistic goal, but by “aiming high” it may be possible to reach what may be a critical threshold for compliance at which the transmission cycle is consistently broken at at least one point (if not several) in every case, so that preventable HAIs do not occur.

The presence of posters only had a significant effect on contact times in clinics where ABHR was not readily available, and for hand hygiene attempts before patient contact or “clean” procedures. As this suggests, a poster campaign of this kind may be more effective in a particular subset of clinics compared to others. Overall the availability of ABHR resulted in longer contact times, independent of whether ABHR or another product was used. It is possible that clinics without ABHR may have a poorer infection control culture, such that the posters were more likely to have an impact on practices. However, neither posters nor availability of ABHR had a detectable effect on compliance. Nonetheless, based on the follow-up survey responses it is clear that the majority of staff noticed the posters, and many reported a self-perceived impact of the posters on their awareness and practices, even though this was not objectively detected. Posters of this kind can contribute to improved infection control culture within a clinic and could potentially be a useful component of a multimodal intervention in conjunction with other items. Recommendations for improving hand hygiene compliance in human healthcare call for multimodal interventions that address the issue in multiple ways, and critically include the involvement and visible support of upper management [1,2,12,23,31,51,52]. Convincing clinics to implement such an intervention with the current lack of infection control culture in veterinary medicine may be difficult. Shea and Shaw [9] reported a 21% increase in hand hygiene compliance at a companion animal teaching hospital following a multimodal educational campaign emphasizing use of a foaming ABHR product. Although it was not possible to discern the effect of the campaign from that of improved availability of ABHR (most of the increase in hand hygiene was due to use of the foam), or from the potential impact of the presence of live observers, given the low use of ABHR in the current study, such a campaign could potentially be effective in other veterinary clinics as well.

The use of video observation in this study had both advantages and disadvantages. Staff behaviours were directly observed, rather than relying on self-reported behaviour from an interview or survey. The cameras allowed for discrete observation of staff compared to direct “live” observation; however, the cameras were visible to staff, and all staff were made aware of the study in advance to provide consent, therefore Hawthorne effects still could have resulted in altered (i.e. artificially improved) behaviour [53]. Recording day was found to have a small but significant positive effect on hand hygiene compliance. It was initially hypothesized that if the recording day had any effect it would be negative, as a result of progressive desensitization to the presence of the cameras (i.e. decreased Hawthorne effects). The appointments coded from each clinic were primarily from the latter two-thirds of the total recording period, at which point it was hoped that most staff would have had at least several days to become acclimatized to the presence of the cameras and resume their typical routine. The cause of this slight positive effect is unclear. No effect of time was seen in a previous study of preoperative preparation practices in veterinary clinics using the same camera system [54]. A constant static effect of the presence of the cameras also cannot be ruled out. The fixed camera positions, which provided a somewhat limited and at times obstructed view, and periodic problems with recording due to signal, power or computer issues likely decreased observational sensitivity. At the same time, the level of detailed video review, facilitated by the ability to watch and re-watch video segments in real time or slow motion as needed, likely increased observational specificity. Due to the complexity of the coding scheme and the time required to code such a large volume of video footage, all videos were ultimately coded by one author (MA). Although verification of all coding by a second observer could have been beneficial to help ensure accuracy, having a single coder who was fully dedicated to the project and who had seen the physical layout of each clinic during site visits likely provided the most consistent and accurate application of the coding scheme across all clinics. There is always potential for some level of observer bias, but the level of detail included in the scheme helped to minimize this. Ideally the observer would have also been blinded with regard to whether or not the posters were in place at the time of each appointment, but this was not possible as the posters were often within the field of view.

Other limitations of this study should also be considered. Clinics were not randomly selected, and participated on a voluntary basis. It is possible that clinic staff with a greater interest in infection control or who were more comfortable with their current practices would be more willing to participate. This would be expected to bias the results toward increased hand hygiene compliance, which is concerning given the low compliance observed. Only primary care clinics in Ontario were included, therefore generalization of results to larger clinics (e.g. tertiary referral hospitals) and those in other regions must be done with caution, if at all. The large number of observations, particularly in the compliance model, increased the likelihood of identifying biologically insignificant effects and interactions as statistically significant. Also, because overall contact times were fairly short, even some of the larger ratios between variables in the contact time model may represent an absolute difference of only a few seconds. Therefore, the statistical models should be viewed as a relevant overview of some of the factors involved and their relative effects, without overemphasizing small differences between categories, even if they are statistically significant.

## Conclusions

This study provided a unique opportunity for detailed observation of hand hygiene practices of staff in companion animal veterinary clinics in Ontario. Overall hand hygiene compliance was low, but not dissimilar from previous reports of hand hygiene compliance in human healthcare facilities. In particular, hand hygiene prior to patient contact needs to be emphasized, as well as attention to performing hand hygiene prior to leaving the patient contact room/area in order to reduce the risk of more distant cross-contamination. Many of the same barriers to hand hygiene likely exist in both the human and veterinary professions, and veterinary clinics may be able to apply some of the lessons learned in human healthcare to improve hand hygiene compliance. The results of this study suggest that, as has been found in human medicine, promoting the use of ABHR in veterinary clinics could potentially improve hand hygiene compliance in terms of timing and technique, as ABHR can easily be made available in any area and in this study was associated with longer product contact time (though still shorter than recommended) compared to soap and water. Many clinics already have ABHR available in some areas, but veterinary personnel need to take more advantage of the benefits and convenience such products offer. The poster campaign had a limited effect on hand hygiene product contact time, with no detectable effect on compliance, but did “connect” with some staff and could potentially be a useful component of a multimodal campaign to improve hand hygiene practices. However, improving the infection control culture in veterinary medicine will likely be required before, or at least concurrent to, any such initiative, so that clinics will be willing to put in the required time and effort to execute a successful campaign.

## Abbreviations

HAI: Hospital-associated infection; ABHR: Alcohol-based hand rub; WHO: World Health Organization; CI: Confidence interval; OR: Odds ratio.

## Competing interests

The authors declare that they have no competing interests.

## Authors’ contributions

MA and JW designed the study. On-site data collection (including camera set up) was performed by MA. MA designed and tested the video coding scheme with input from JW. All videos were coded and data cleaning was performed by MA. Analysis was performed by MA with input from JS. The manuscript was drafted by MA and revised by JW and JS. All authors read and approved the final manuscript.

## Supplementary Material

Additional file 1 Poster A, used as part of an intervention to help improve hand hygiene compliance among staff in companion animal veterinary clinics in Ontario, which was mounted in exam rooms (actual size 22 cm x 28 cm). Click here for file

Additional file 2 Poster B, used as part of an intervention to help improve hand hygiene compliance among staff in companion animal veterinary clinics in Ontario, which was mounted in backroom areas (actual size 22 cm x 28 cm). Click here for file

Additional file 3 **Justification for the key elements included on Posters A and B (see Additional files**1**&**2**) designed to help improve hand hygiene compliance among staff in companion animal veterinary clinics in Ontario.** Click here for file

Additional file 4 Information coded at the clinic, appointment, individual and hand hygiene opportunity level from video footage. Click here for file

Additional file 5 Additional details of video coding scheme used to measure hand hygiene compliance in companion animal veterinary clinics. Click here for file

Additional file 6 Descriptive data for veterinary clinics included in the analysis for the hand hygiene intervention trial. Click here for file

Additional file 7 Details of hand hygiene opportunities and attempts observed during 2278 companion animal veterinary appointments. Click here for file

Additional file 8 All contrasts of associations for variables included in the final multivariable random effects logistic regression model for observed hand hygiene compliance for opportunities associated with routine companion animal appointments at 38 veterinary clinics in Ontario (n = 10894). Click here for file

Additional file 9 Histogram and normal quantile plot for residuals of the final multivariable random effects linear regression model for product contact time at the sample level (hand hygiene attempt) after log transformation of the outcome. Click here for file

Additional file 10 All contrasts of associations for variables included in the final multivariable random effects linear regression model for product contact time for hand hygiene attempts associated with routine companion animal appointments at 38 veterinary clinics in Ontario (n = 1330). Click here for file
