# Supplementary material for: Video observation of hand hygiene practices during routine companion animal appointments and the effect of a poster intervention on hand hygiene compliance
Source: BMC Vet Res. 2014 May 7;10:106. doi: 10.1186/1746-6148-10-106 (PMC4108058; doi:10.1186/1746-6148-10-106)
Supplement: Additional file 1 — Poster A, used as part of an intervention to help improve hand hygiene compliance among staff in companion animal veterinary clinics in Ontario, which was mounted in exam rooms (actual size 22 cm x 28 cm). [file 1746-6148-10-106-S1.pdf]

Pets can carry infections too!

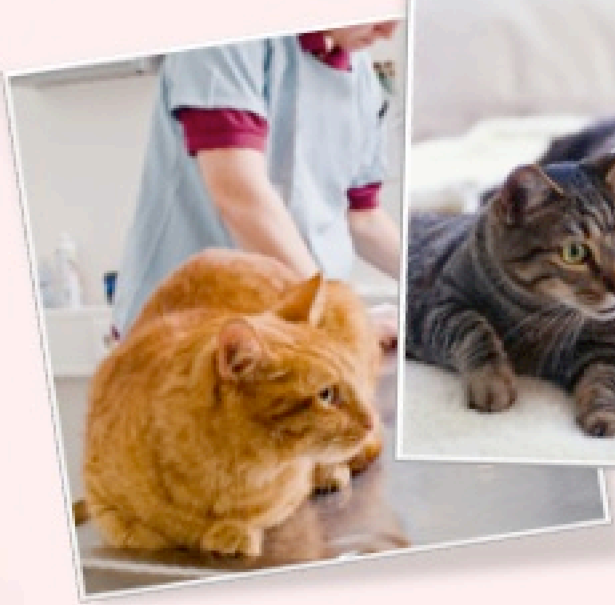

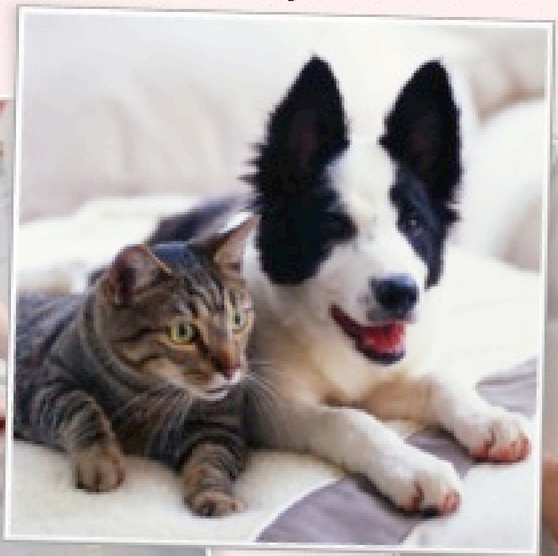

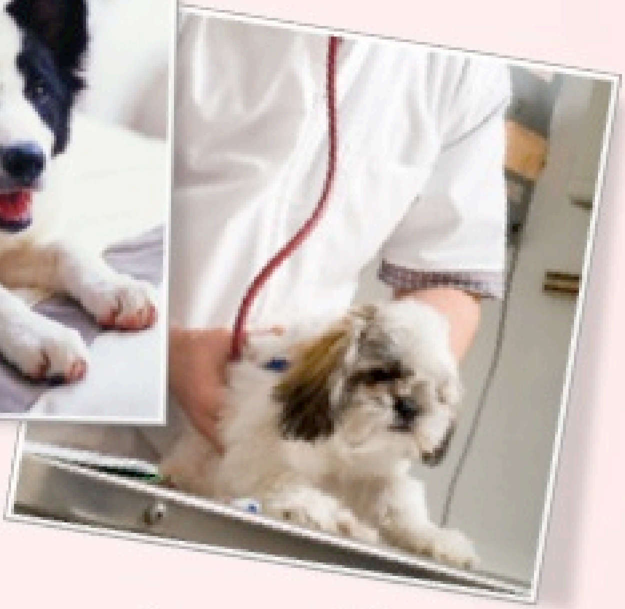

Protect pets, their owners and yourself:

PAUSE TO CLEAN YOUR PAWS!

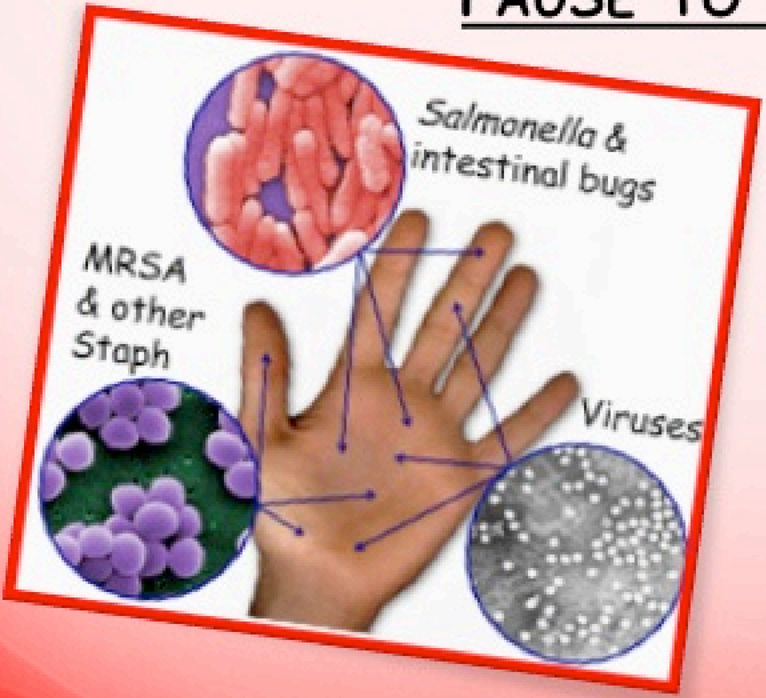

Hands are the #1 way infections are spread.

It only takes 15 seconds to clean your hands.
Wash your hands or use an alcohol-based hand sanitizer to help stop the spread of infections
to pets,
to their owners,
and to you!

Clean Hands =
Healthier Pets + Healthier People
