# Supplementary material for: Video observation of hand hygiene practices during routine companion animal appointments and the effect of a poster intervention on hand hygiene compliance
Source: BMC Vet Res. 2014 May 7;10:106. doi: 10.1186/1746-6148-10-106 (PMC4108058; doi:10.1186/1746-6148-10-106)
Supplement: Additional file 2 — Poster B, used as part of an intervention to help improve hand hygiene compliance among staff in companion animal veterinary clinics in Ontario, which was mounted in backroom areas (actual size 22 cm x 28 cm). [file 1746-6148-10-106-S2.pdf]

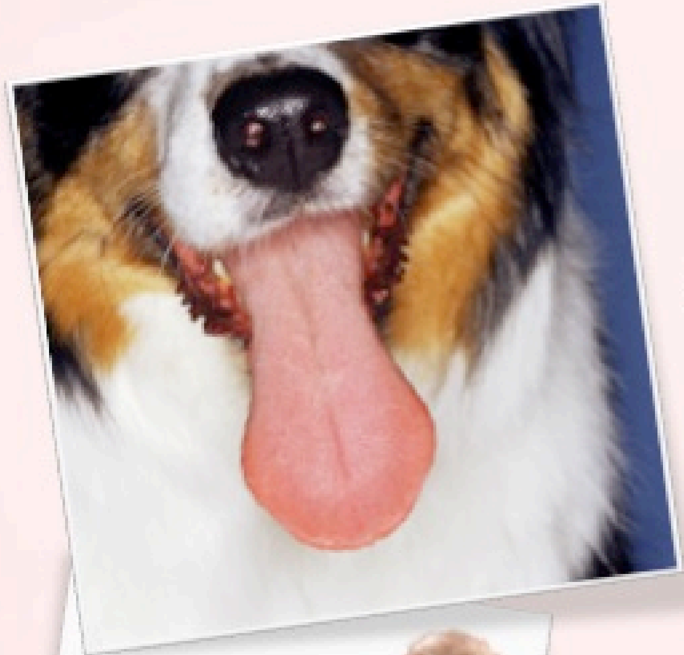

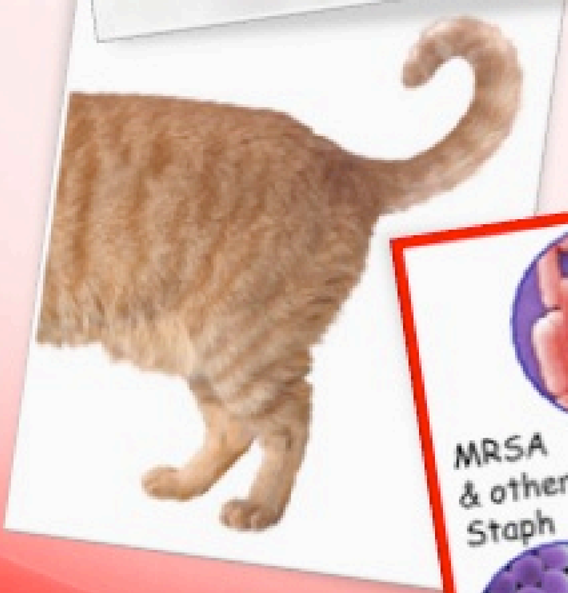

If you could see what's there...

...you'd CLEAN what's here!

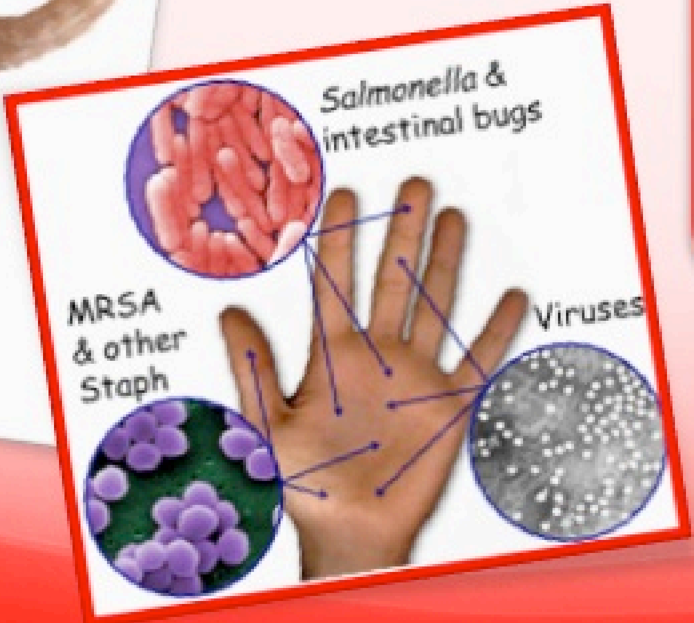

Protect pets *and* people:

PAUSE TO CLEAN YOUR PAWS!

Pets can carry a large number of bacteria, viruses and parasites in their mouths, noses, skin and fur – often with no signs of illness or infection.

Many can be transmitted to other animals, and some even to people!

Hands are the #1 way infections spread. **It only takes 15 seconds to clean your hands properly** by washing or using an alcohol-based hand sanitizer.
It can help stop the spread of infections
to pets,
to their owners,
and to you!

Clean Hands = Healthier Pets + Healthier People
