# Supplementary material for: Video observation of hand hygiene practices during routine companion animal appointments and the effect of a poster intervention on hand hygiene compliance
Source: BMC Vet Res. 2014 May 7;10:106. doi: 10.1186/1746-6148-10-106 (PMC4108058; doi:10.1186/1746-6148-10-106)
Supplement: Additional file 3 — Justification for the key elements included on Posters A and B (see Additional files1&2) designed to help improve hand hygiene compliance among staff in companion animal veterinary clinics in Ontario. [file 1746-6148-10-106-S3.pdf]

Additional file 3: Justification for the key elements included on Posters A and B [see Additional Files 1 & 2) designed to help improve hand hygiene compliance among staff in companion animal veterinary clinics in Ontario

Poster theory[1]	Element of hand hygiene posters
Avoid making the poster a “training chart,” as knowledge does not necessarily affect behavioural change in a positive direction.	Posters do NOT show how to wash hands, but rather focus on why to wash hands.
Most health professionals (which may include veterinary staff in this case) know/believe/are aware that hand hygiene can prevent cross contamination, however, based on poor compliance it is thought that there is low “personal acceptance” of this fact, meaning that many health professionals do not believe it is applicable specifically to themselves. To motivate individuals to perform hand hygiene they need to believe it is effective (i.e. for preventing cross contamination) and that they themselves are at risk.	Emphasis that “hands are the #1 way infections are spread” and picture showing multiple pathogens that can be carried on the hands reinforces the utility of hand hygiene for preventing pathogen spread.
	Include reminders of personal benefits (“protect...yourself,” “stop spread of infections...to you”)
In the case of health professionals, who themselves may be relatively fit and healthy and at reasonably low risk of infection, it also important to appeal to their sense of obligation to protect others (i.e. patients and clients).	“Protect pets, their owners…”<br>“Protect pets and people”
	“stop spread of infections to pets, to their owners…”
It is useful to emphasize minimization of losses/barriers (such as time) so individuals realize that they not only should perform hand hygiene but that they can.	“It only takes 15 seconds to clean your hands…” in bold.
Gain-framing (versus loss-framing) has been shown to be more effective for promoting prevention behaviors.	Emphasis on “stop spread of infection” by using hand hygiene, rather than “infections may occur” if hand hygiene is not used.
	Emphasis on “protect.”
	“Clean hands = Healthier pets + Healthier people” emphasizes gains of hand hygiene.
Fear-appeals can backfire (especially if moderate to severe) and must be related to personal concern to be effective (e.g. self-infection).	Fear appeals avoided except on Poster B, where they are limited to pointing out that infections “can be transmitted to other animals, and some even to people” which is related to personal (self) concern.
One size does not fit all.	Two slightly different posters used (Poster A in exam rooms, Poster B in backroom areas).

[1] Based on (Jenner E, et al. Hand hygiene posters: selling the message. J Hosp Infect. 2005;59(2):77–82.)
