# Supplementary material for: Video observation of hand hygiene practices during routine companion animal appointments and the effect of a poster intervention on hand hygiene compliance
Source: BMC Vet Res. 2014 May 7;10:106. doi: 10.1186/1746-6148-10-106 (PMC4108058; doi:10.1186/1746-6148-10-106)
Supplement: Additional file 4 — Information coded at the clinic, appointment, individual and hand hygiene opportunity level from video footage. [file 1746-6148-10-106-S4.pdf]

Additional file 4: Information coded at the clinic, appointment, individual and hand hygiene opportunity level from video footage

Clinic level variables	Appointment level variables	Individual level variables	HH opportunity level variables
-Clinic number<br>-Sink present in exam room (yes/no)<br>-ABHR readily available in clinic (yes/no)	-Appointment number<br>-Appointment type (vaccine only/ vaccine and ≥ 1 other clean or dirty procedure/other)<br>-Patient species (dog/cat/other/ multiple)<br>-Posters present (yes/no)<br>-Recording day (measured as the number of working days before or after the posters were placed in the clinic (= day 0))	-Individual number<br>-Role (veterinarian/ technician/ other)<br>-Gender (male/female)	-Opportunity number<br>-Room[1] (exam room/backroom)<br>-Opportunity type (1-5)[2]<br>-Unique vs coincident opportunity (yes/no)<br>-Facial contact prior to HH[3] (yes/no)<br>-HH timing (0-5)[4]<br>-Timing of glove removal (if applicable) (0-5)[4]<br>-HH product used (water/soap-&-water/ABHR)<br>-HH product contact time (in seconds)<br>-Hand jewelry (none/watch/ring/bracelet/multiple)<br>-Visible deliberate effort to scrub/rub (yes/no/not visible):<br>>back of hand(s)<br>>between fingers<br>>thumb(s)<br>>wrist(s)<br>-Contact with water faucet after HH (none/disposable towel/hand/arm or elbow)<br>-Hand drying technique (none/disposable towel/reusable towel/clothes/shaking hands)

HH=hand hygiene, ABHR=alcohol-based hand rub

[1] the room in which the animal contact occurred, resulting in a hand hygiene opportunity, not necessarily the room in which hand hygiene was attempted

[2] 1: before animal contact, 2: before a “clean” procedure (without or without gloves), 3: after a “dirty” procedure without gloves, 4: after glove removal, 5: after animal contact

[3] for hand hygiene opportunities after exposure/procedure/contact; this included indirect facial contact with the animal via an individual touching (with a hand) any part of his/her own face forward of the ears as well as touching hair that was likely to have contact with the same area, and direct face-to-animal contact (e.g. kissing, licking, snuggling, contact with the face during restraint)

[4] 0: unobserved, 1: not performed prior to next “clean” procedure, contact with a “cleaner” part of the same animal, or contact with an unrelated animal, 2: outside of room having touched other objects/surfaces, 3: outside of room without having touched other objects/surfaces, 4: in room having touched other objects/surfaces not in direct contact with animal, 5: in room without having touched other objects/surfaces not in direct contact with animal
