# Supplementary material for: Video observation of hand hygiene practices during routine companion animal appointments and the effect of a poster intervention on hand hygiene compliance
Source: BMC Vet Res. 2014 May 7;10:106. doi: 10.1186/1746-6148-10-106 (PMC4108058; doi:10.1186/1746-6148-10-106)
Supplement: Additional file 5 — Additional details of video coding scheme used to measure hand hygiene compliance in companion animal veterinary clinics. [file 1746-6148-10-106-S5.pdf]

Additional file 5: Additional details of video coding scheme used to measure hand hygiene compliance in companion animal veterinary clinics

A complete "contact cycle" was considered observation of an individual's initial contact and final contact with a patient, before being off camera for over 2 min or having visible contact with an unrelated animal. For hand hygiene opportunities before patient contact, or before "clean" procedures, only hand hygiene attempts made within the same room/area as the applicable contact were coded (i.e. hand hygiene timing was only coded as 1, 4, or 5, see Table 2). If an individual left the room/area where the last animal contact took place, but did not leave the field of view for more than 2 min or have visible contact with another animal before returning, this was considered part of the same contact cycle, but any subsequent hand hygiene attempt for "after contact" was coded as "outside of room having touched other objects/surfaces," even if it was performed in the room upon the person's return. If an individual left the room/area where the last animal contact took place, and then left the field of view for more than 2 min without first attempting hand hygiene, hand hygiene timing for "after contact" was coded as "unobserved" and a new contact cycle was coded if the individual returned and had further contact with the animal, beginning with an additional "before contact" hand hygiene opportunity. This 2-minute interval was based on the possibility of the individual having contact with either another animal or a sufficient number/type of objects/surfaces while off-camera over a period of 2 min or more to warrant an additional hand hygiene attempt prior to subsequent patient contact. If the person was off-camera for less than 2 min then the likelihood of such contact was lower, and the benefit of the doubt was given that additional hand hygiene prior to subsequent patient contact would not be as useful.

If hand hygiene was not observed for an after contact/procedure/degloving opportunity (i.e. hand hygiene opportunity type 3, 4, or 5, see Table 2), additional information coded included whether the individual was seen to touch any other objects/surfaces outside of the patient contact room/area before leaving the field of view. Individuals were followed for up to 2 min after leaving an appointment or last having contact with the patient in the backroom, if the person remained in the field of view for this long. If the

person left the field of view in less than 2 min and was off-camera for more than 20 s (in clinics with AHS available) or more than 30s (in clinics without AHS available), then hand hygiene was coded as "unobserved, left view." If the individual was in view for at least 2 min without going off-camera for more than 20-30 s (depending on the clinic), then hand hygiene was coded as "unobserved, stayed in view." This 2-minute interval was selected to increase the likelihood that any hand hygiene attempt coded was related primarily to the appointment/relevant animal contact rather than other tasks performed subsequently. An exception to this interval was made for staff who remained in the field of view and were clearly completing medical records, without engaging in any other tasks, in which case they were followed for up to 5 min to observe a hand hygiene attempt. The 20 s interval was the estimated minimum amount of time that would be required to reach, use and return from an AHS station located in another area of the clinic, while the 30 s interval was the same for a hand washing station (i.e. sink with soap and water). If an individual performed a "dirty" procedure or removed gloves prior to leaving the field of view, and was then off camera for less than 2 min but for at least 20-30 s (depending on the clinic), hand hygiene timing for that opportunity was coded as unobserved, because a hand hygiene attempt could not be excluded if the individual was off-camera for this interval (and otherwise subsequent contact with a "clean" part of the patient (without performing hand hygiene) would be considered a "failure" (i.e. hand hygiene timing = 1, not performed)).

If a person entered or left the field of view in direct contact with a patient (including leading a dog on leash, but not including carrying a cat in a closed carrier) and was off-camera for at least 20-30 s (depending on the clinic, i.e. long enough to potentially perform hand hygiene in an unmonitored area) prior to or after being seen with the animal, then no hand hygiene opportunity was coded. Contact with multiple animals brought in by the same client in the same appointment was treated as contact with a single animal, but if the same procedure (e.g. vaccination) was performed on more than one of these animals consecutively, these were considered different procedures and a hand hygiene opportunity was coded for each, if applicable.
