# Supplementary material for: Video observation of hand hygiene practices during routine companion animal appointments and the effect of a poster intervention on hand hygiene compliance
Source: BMC Vet Res. 2014 May 7;10:106. doi: 10.1186/1746-6148-10-106 (PMC4108058; doi:10.1186/1746-6148-10-106)
Supplement: Additional file 6 — Descriptive data for veterinary clinics included in the analysis for the hand hygiene intervention trial. [file 1746-6148-10-106-S6.pdf]

Additional file 6: Descriptive data for veterinary clinics included in the analysis for the hand hygiene intervention trial

Clinic	Estimated number of staff	Number of staff coded	Total appts coded	Mean appts per day	Total HH opps coded	Mean HH opps per appt	% HH compl[1]	Backroom camera in clinical area	Sink in exam room	ABHR readily available in clinic
1	12	8	20	7	70	4	9	No	No	Yes
2	8	7	20	2	100	5	12	Yes	No	No
3	4	4	22	2	104	5	17	Yes	Yes	No
4	8	7	26	3	144	6	10	Yes	Yes	No
5	16	16	30	3	244	8	14	Yes	Yes	No
6	13	11	30	5	149	5	7	No	No	Yes
7	12	11	32	3	163	5	8	Yes	Yes	Yes
8	7	7	34	2	157	5	7	Yes	Yes	Yes
9	6	6	34	4	268	8	7	No	Yes	Yes
10	4	4	40	3	282	7	24	Yes	Yes	No
11	11	11	42	3	271	6	12	Yes	Yes	Yes
12	5	5	44	4	142	3	12	Yes	Yes	No
13	18	15	54	4	275	5	17	No	Yes	Yes
14	11	11	54	4	281	5	17	Yes	Yes	Yes
15	12	10	56	4	299	5	11	Yes	Yes	No
16	9	9	60	6	234	4	11	Yes	No	Yes
17	13	11	62	4	207	3	26	No	Yes	No
18	5	5	66	4	221	3	24	Yes	No	No
19	49	39	66	6	365	6	14	No	Yes	Yes
20	12	11	68	5	250	4	1	Yes	No	Yes
21	7	6	72	6	204	3	17	No	No	Yes
22	12	8	74	6	351	5	9	Yes	No	No
23	14	14	74	6	287	4	18	Yes	Yes	Yes
24	21	20	78	8	402	5	10	Yes	Yes	No
25	12	10	80	9	289	4	17	No	Yes	Yes
26	23	20	80	9	631	8	22	Yes	Yes	Yes
27	18	10	80	7	369	5	19	No	Yes	Yes
28	9	9	80	8	342	4	9	Yes	No	Yes
29	9	9	80	9	441	6	10	No	Yes	Yes
30	15	15	80	10	304	4	13	Yes	Yes	Yes
31	15	12	80	7	370	5	28	No	Yes	Yes
32	15	13	80	8	337	4	16	No	No	Yes
33	22	16	80	7	379	5	4	No	No	Yes
34	11	10	80	7	424	5	10	Yes	Yes	Yes
35	11	11	80	6	320	4	10	No	No	No
36	15	14	80	11	377	5	16	No	Yes	Yes
37	22	21	80	20	539	7	6	Yes	Yes	Yes
38	24	23	80	8	302	4	11	No	No	Yes
**Total**	**504**	**449**	**2278**	**-**	**10894**	**-**	**-**	**-**	**-**	**-**
**Mean**	**13**	**12**	**60**	**6**	**287**	**5**	**13**	**-**	**-**	**-**
**Median**	**12**	**11**	**67**	**6**	**285**	**5**	**12**	**-**	**-**	**-**
**Range**	**4-49**	**4-39**	**20-80**	**2-20**	**70-631**	**3-8**	**1-28**	**-**	**-**	**-**

appts=appointments, HH opps=hand hygiene opportunities, ABHR=alcohol-based hand rub

[1] % HH compl = hand hygiene compliance = HH opps with observed attempt / total HH opps x100
