# Supplementary material for: Video observation of hand hygiene practices during routine companion animal appointments and the effect of a poster intervention on hand hygiene compliance
Source: BMC Vet Res. 2014 May 7;10:106. doi: 10.1186/1746-6148-10-106 (PMC4108058; doi:10.1186/1746-6148-10-106)
Supplement: Additional file 7 — Details of hand hygiene opportunities and attempts observed during 2278 companion animal veterinary appointments. [file 1746-6148-10-106-S7.pdf]

Additional file 7: Details of hand hygiene opportunities and attempts observed during 2278 companion animal veterinary appointments

		Hand hygiene attempt observed			
Hand hygiene opportunity type	n	Percent in same room/area (n)	Percent in other room/area (n)	Percent not performed (n)	Percent not observed[f] (n)
Before patient contact	4377	3 (114)	0.2 (9) [b]	96 (4220) [c]	1 (34)
Before a "clean" procedure[a]	1524	2 (24)	0.1 (1) [b]	98 (1497) [c]	0.1 (2)
After a "dirty" procedure without gloves[a]	463	15 (69)	11 (51)	51 (235) [d]	23 (108)
After glove removal	153	30 (46)	9 (14)	39 (59) [d]	22 (34)
After patient contact	4377	8 (350)	18 (795)	3 (112) [e]	71 (3120)
Total	10894	6 (603)	8 (870)	56 (6123)	30 (3298)

[a] see Table 1 for list of procedures considered "clean" vs "dirty"

[b] in these cases, the individual performed hand hygiene in the exam room at the start of the appointment, but then left the room briefly (< 2 min) and returned prior to touching the animal for the first time (4) or performing an injection (1), or the individual performed hand hygiene in the exam room and then exited to front office and returned with a client and animal in < 60 s to start the appointment (5)

[c] no hand hygiene attempt was made within the same room prior to contact/procedure

[d] no hand hygiene attempt was observed prior to a "clean" procedure, contact with a "clean" part of the same animal, or contact with an unrelated animal, and the individual was not off-camera for a sufficient duration (20-30s) to perform hand hygiene elsewhere

[e] no hand hygiene attempt was observed prior to contact with an unrelated animal and the individual was not off-camera for a sufficient duration (20-30s) to perform hand hygiene elsewhere

[f] no hand hygiene attempt was observed in the room where the contact/procedure took place, and no attempt was observed within 2 min of leaving the original room/area or before the individual went off-camera for a sufficient duration (20-30s) to perform hand hygiene elsewhere
