# Supplementary material for: Video observation of hand hygiene practices during routine companion animal appointments and the effect of a poster intervention on hand hygiene compliance
Source: BMC Vet Res. 2014 May 7;10:106. doi: 10.1186/1746-6148-10-106 (PMC4108058; doi:10.1186/1746-6148-10-106)
Supplement: Additional file 8 — All contrasts of associations for variables included in the final multivariable random effects logistic regression model for observed hand hygiene compliance for opportunities associated with routine companion animal appointments at 38 veterinary clinics in Ontario (n = 10894). [file 1746-6148-10-106-S8.pdf]

Additional file 8: All contrasts of associations for variables included in the final multivariable random effects logistic regression model for observed hand hygiene compliance for opportunities associated with routine companion animal appointments at 38 veterinary clinics in Ontario (n = 10894)

Variable	Interaction	Comparison	OR	95% CI		p-value
				Lower	Upper	
Posters	-	Absent vs present	1.04	0.91	1.19	0.5347
**Recording day**	**-**	**Per 1 day increase**	**1.04**	**1.002**	**1.087**	**0.0408**
Room*role	Exam room	**Other vs veterinarian**	**0.27**	**0.19**	**0.39**	**<0.0001**
		**Other vs technician**	**0.58**	**0.40**	**0.85**	**0.0053**
		**Veterinarian vs technician**	**2.15**	**1.80**	**2.56**	**<0.0001**
	Backroom	**Other vs veterinarian**	**0.21**	**0.05**	**0.94**	**0.0408**
		**Other vs technician**	**0.19**	**0.04**	**0.83**	**0.0271**
		Veterinarian vs technician	0.90	0.63	1.28	0.5522
	Exam room vs backroom	Other	0.72	0.15	3.34	0.6723
		**Veterinarian**	**0.56**	**0.38**	**0.83**	**0.0036**
		**Technician**	**0.23**	**0.15**	**0.35**	**<0.0001**
Gender*HH opp type	Male	**Before patient contact vs before "clean" procedure**	**9.16**	**2.02**	**41.56**	**0.0041**
		**Before patient contact vs after "dirty" procedure**	**0.10**	**0.05**	**0.19**	**<0.0001**
		**Before patient contact vs after glove removal**	**0.10**	**0.04**	**0.24**	**<0.0001**
		**Before patient contact vs after patient contact**	**0.14**	**0.09**	**0.22**	**<0.0001**
		**Before "clean" procedure vs after "dirty" procedure**	**0.01**	**0.002**	**0.05**	**<0.0001**
		**Before "clean" procedure vs after glove removal**	**0.01**	**0.002**	**0.06**	**<0.0001**
		**Before "clean" procedure vs after patient contact**	**0.02**	**0.004**	**0.07**	**<0.0001**
		After "dirty" procedure vs after glove removal	1.08	0.43	2.68	0.8761
		After "dirty" procedure vs after patient contact	1.51	0.85	2.69	0.1575
		After glove removal vs after patient contact	1.41	0.65	3.03	0.3824
		Before patient contact vs before "clean" procedure	1.40	0.81	2.42	0.2263

Variable	Interaction	Comparison	OR	95% CI		p-value
				Lower	Upper	
Gender*HH opp type	Female	**Before patient contact vs after "dirty" procedure**	**0.05**	**0.03**	**0.08**	**<0.0001**
		**Before patient contact vs after glove removal**	**0.03**	**0.02**	**0.06**	**<0.0001**
		**Before patient contact vs after patient contact**	**0.07**	**0.05**	**0.09**	**<0.0001**
		**Before "clean" procedure vs after "dirty" procedure**	**0.04**	**0.02**	**0.07**	**<0.0001**
		**Before "clean" procedure vs after glove removal**	**0.02**	**0.01**	**0.05**	**<0.0001**
		**Before "clean" procedure vs after patient contact**	**0.05**	**0.03**	**0.08**	**<0.0001**
		After "dirty" procedure vs after glove removal	0.68	0.37	1.26	0.2172
		After "dirty" procedure vs after patient contact	1.40	0.93	2.09	0.1052
		**After glove removal vs after patient contact**	**2.06**	**1.23**	**3.43**	**0.0057**
	Male vs female	Before patient contact	1.21	0.79	1.83	0.3784
		**Before "clean" procedure**	**0.18**	**0.04**	**0.80**	**0.0244**
		After "dirty" procedure	0.62	0.37	1.05	0.0749
		**After glove removal**	**0.39**	**0.18**	**0.88**	**0.0224**
		**After patient contact**	**0.57**	**0.46**	**0.71**	**<0.0001**
Room*HH opp type	Exam room	**Before patient contact vs before "clean" procedure**	**4.12**	**1.91**	**8.90**	**0.0003**
		**Before patient contact vs after "dirty" procedure**	**0.12**	**0.09**	**0.17**	**<0.0001**
		**Before patient contact vs after glove removal**	**0.08**	**0.05**	**0.13**	**<0.0001**
		**Before patient contact vs after patient contact**	**0.08**	**0.07**	**0.10**	**<0.0001**
		**Before "clean" procedure vs after "dirty" procedure**	**0.03**	**0.01**	**0.07**	**<0.0001**
		**Before "clean" procedure vs after glove removal**	**0.02**	**0.008**	**0.04**	**<0.0001**
		**Before "clean" procedure vs after patient contact**	**0.02**	**0.01**	**0.04**	**<0.0001**
		After "dirty" procedure vs after glove removal	0.63	0.38	1.03	0.0669

Variable	Interaction	Comparison	OR	95% CI		p-value
				Lower	Upper	
Room*HH opp type	Exam room	**After “dirty” procedure vs after patient contact**	**0.67**	**0.51**	**0.89**	**0.0054**
		After glove removal vs after patient contact	1.07	0.69	1.66	0.7631
	Backroom	Before patient contact vs before “clean” procedure	3.11	0.95	10.21	0.0614
		**Before patient contact vs after “dirty” procedure**	**0.04**	**0.02**	**0.09**	**<0.0001**
		**Before patient contact vs after glove removal**	**0.04**	**0.02**	**0.12**	**<0.0001**
		**Before patient contact vs after patient contact**	**0.12**	**0.07**	**0.20**	**<0.0001**
		**Before “clean” procedure vs after “dirty” procedure**	**0.01**	**0.003**	**0.05**	**<0.0001**
		**Before “clean” procedure vs after glove removal**	**0.01**	**0.004**	**0.06**	**<0.0001**
		**Before “clean” procedure vs after patient contact**	**0.04**	**0.01**	**0.12**	**<0.0001**
		After “dirty” procedure vs after glove removal	1.17	0.39	3.45	0.07818
		**After “dirty” procedure vs after patient contact**	**3.15**	**1.49**	**6.70**	**0.0028**
		**After glove removal vs after patient contact**	**2.71**	**1.13**	**6.50**	**0.0261**
	Exam room vs backroom	Before patient contact	0.64	0.31	1.29	0.2119
		Before “clean” procedure	0.48	0.16	1.42	0.1863
		**After “dirty” procedure**	**0.19**	**0.08**	**0.48**	**0.0004**
		After glove removal	0.36	0.13	1.03	0.0574
		After patient contact	0.91	0.54	1.54	0.7278

OR=odds ratio, CI=confidence interval, HH opp=hand hygiene opportunity
Statistically significant contrasts ($p<0.05$) are in **boldface**
