# Supplementary material for: Video observation of hand hygiene practices during routine companion animal appointments and the effect of a poster intervention on hand hygiene compliance
Source: BMC Vet Res. 2014 May 7;10:106. doi: 10.1186/1746-6148-10-106 (PMC4108058; doi:10.1186/1746-6148-10-106)

Additional file 9: Histogram and normal quantile plot for residuals of the final multivariable random effects linear regression model for product contact time at the sample level (hand hygiene attempt) after log transformation of the outcome

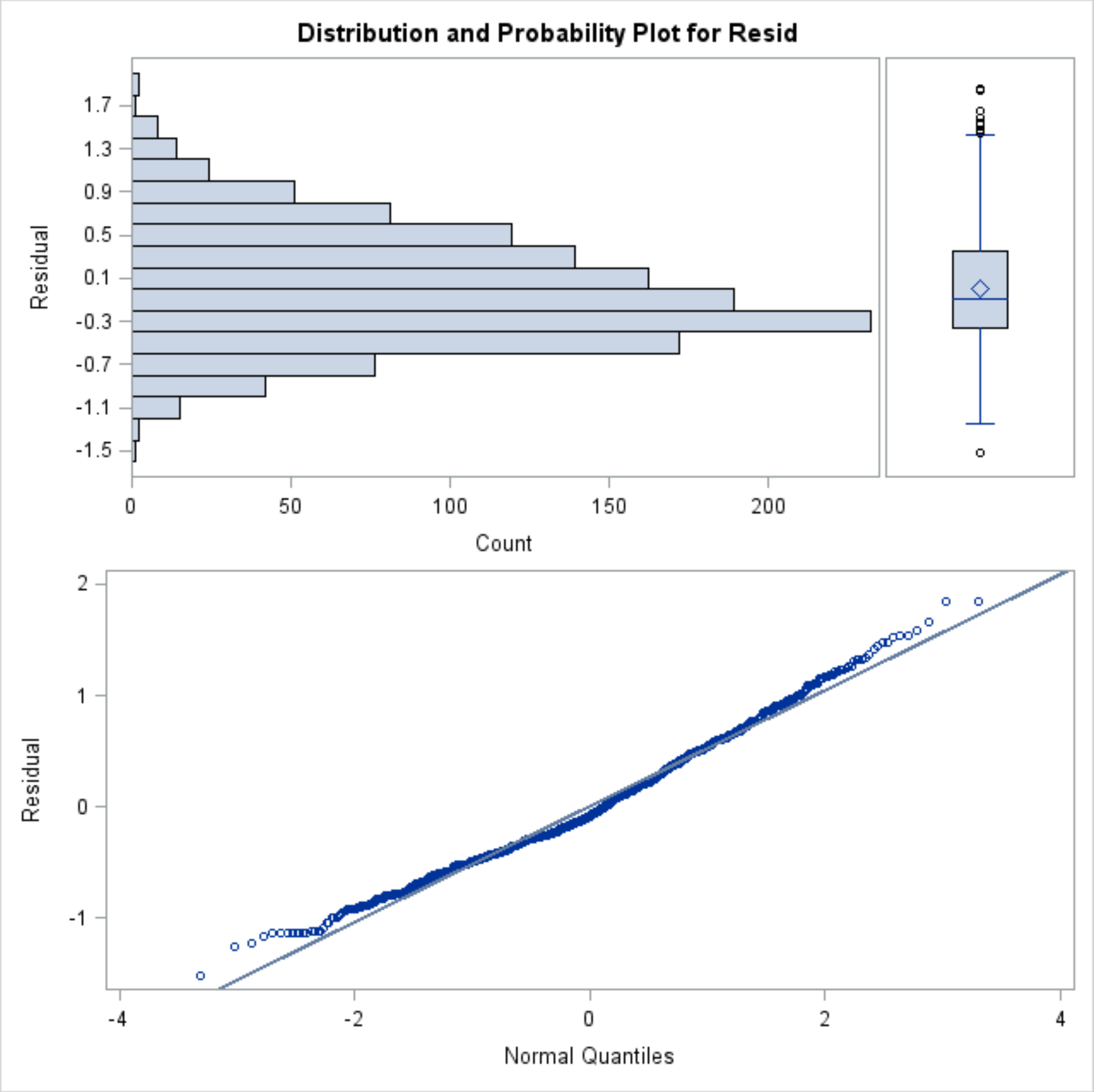

Supplement: Additional file 9 — Histogram and normal quantile plot for residuals of the final multivariable random effects linear regression model for product contact time at the sample level (hand hygiene attempt) after log transformation of the outcome. [file 1746-6148-10-106-S9.pdf]
