# Supplementary material for: Video observation of hand hygiene practices during routine companion animal appointments and the effect of a poster intervention on hand hygiene compliance
Source: BMC Vet Res. 2014 May 7;10:106. doi: 10.1186/1746-6148-10-106 (PMC4108058; doi:10.1186/1746-6148-10-106)
Supplement: Additional file 10 — All contrasts of associations for variables included in the final multivariable random effects linear regression model for product contact time for hand hygiene attempts associated with routine companion animal appointments at 38 veterinary clinics in Ontario (n = 1330). [file 1746-6148-10-106-S10.pdf]

Additional file 10: All contrasts of associations for variables included in the final multivariable random effects linear regression model for product contact time for hand hygiene attempts associated with routine companion animal appointments at 38 veterinary clinics in Ontario (n = 1330)

Variable	Interaction term	Comparison	Ratio	95% CI		p-value
				Lower	Upper	
Gender	-	**Male vs female**	**1.20**	**1.04**	**1.38**	**0.0120**
Species	-	Other vs dog	1.34	0.75	2.39	0.3242
		Other vs cat	1.13	0.63	2.03	0.6725
		Other vs multiple	1.38	0.76	2.51	0.2893
		**Dog vs cat**	**0.85**	**0.75**	**0.96**	**0.0074**
		Dog vs multiple	1.03	0.88	1.21	0.7019
		**Cat vs multiple**	**1.22**	**1.01**	**1.46**	**0.0368**
HH product	-	**Water vs soap**	**0.74**	**0.60**	**0.92**	**0.0066**
		**Water vs AHS**	**0.44**	**0.33**	**0.59**	**<0.0001**
		**Soap vs AHS**	**0.59**	**0.48**	**0.72**	**<0.0001**
AHS in clinic*role	AHS in clinic	**Other vs veterinarian**	**1.82**	**1.37**	**2.44**	**<0.0001**
		Other vs technician	1.19	0.87	1.61	0.2757
		**Veterinarian vs technician**	**0.65**	**0.57**	**0.75**	**<0.0001**
	AHS not in clinic	Other vs veterinarian	0.76	0.51	1.12	0.1665
		Other vs technician	0.71	0.46	1.09	0.1162
		Veterinarian vs technician	0.93	0.73	1.18	0.5409
	AHS not in clinic vs AHS in clinic	Veterinarian	0.85	0.64	1.14	0.2852
		**Technician**	**0.60**	**0.42**	**0.85**	**0.0043**
		**Other**	**0.36**	**0.21**	**0.61**	**0.0002**
Posters*HH opp type	Posters present	Before patient contact vs before "clean" procedure	1.23	0.65	2.33	0.5145
		**Before patient contact vs after "dirty" procedure**	**1.63**	**1.16**	**2.29**	**0.0054**
		**Before patient contact vs after glove removal**	**1.83**	**1.14**	**2.95**	**0.0128**
		**Before patient contact vs after patient contact**	**1.46**	**1.15**	**1.86**	**0.0021**
		Before "clean" procedure vs after "dirty" procedure	1.32	0.69	2.53	0.3981
		Before "clean" procedure vs after glove removal	1.49	0.73	3.04	0.2758
		Before "clean" procedure vs after patient contact	1.19	0.65	2.16	0.5749

Variable	Interaction term	Comparison	Ratio	95% CI		p-value
				Lower	Upper	
Posters*HH opp type	Posters present	After “dirty” procedure vs after glove removal	1.12	0.68	1.86	0.6428
		After “dirty” procedure vs after patient contact	0.90	0.69	1.17	0.4169
		After glove removal vs after patient contact	0.80	0.52	1.23	0.3073
	Posters absent	**Before patient contact vs before “clean” procedure**	**2.32**	**1.36**	**3.97**	**0.0023**
		Before patient contact vs after “dirty” procedure	1.25	0.87	1.77	0.2222
		Before patient contact vs after glove removal	1.04	0.68	1.59	0.8511
		Before patient contact vs after patient contact	1.09	0.89	1.34	0.09051
		Before “clean” procedure vs after “dirty” procedure	0.54	0.30	0.96	0.6223
		**Before “clean” procedure vs after glove removal**	**0.45**	**0.25**	**0.80**	**0.0068**
		**Before “clean” procedure vs after patient contact**	**0.47**	**0.28**	**0.78**	**0.0041**
		After “dirty” procedure vs after glove removal	0.84	0.52	1.34	0.4567
		After “dirty” procedure vs after patient contact	0.88	0.65	1.19	0.4010
		After glove removal vs after patient contact	1.05	0.72	1.54	0.7930
	Posters absent vs posters present	**Before patient contact**	**0.68**	**0.50**	**0.92**	**0.0132**
		**Before “clean” procedure**	**0.36**	**0.17**	**0.79**	**0.0109**
		After “dirty” procedure	0.89	0.60	1.32	0.5691
		After glove removal	1.20	0.68	2.11	0.5210
		After patient contact	0.91	0.82	1.02	0.1007
Posters*AHS in clinic	Posters present	**AHS not in clinic vs AHS in clinic**	**0.64**	**0.46**	**0.89**	**0.0086**
	Posters absent	**AHS not in clinic vs AHS in clinic**	**0.50**	**0.36**	**0.70**	**<0.0001**
	Posters absent vs posters present	AHS in clinic	0.85	0.68	1.07	0.1716
		**AHS not in clinic**	**0.66**	**0.50**	**0.88**	**0.0043**

CI=confidence interval; multiple=more than 1 dog and/or cat; HH=hand hygiene; AHS=alcohol-based hand sanitizer; HH opp=hand hygiene opportunity

Statistically significant contrasts ($p<0.05$) are in **boldface**
